# Mapping Trajectories of Hospice Clinician Presence Throughout the Medical Aid in Dying Procedure

**DOI:** 10.1093/geroni/igaf122.870

**Published:** 2025-12-31

**Authors:** Todd Becker, Karla Washington, Stacy Fischer, Elissa Kozlov, Daniel Matlock, Denae Gerasta, Grant Yoder

**Affiliations:** WashU Medicine, St. Louis, Missouri, United States; Washington University in St. Louis, St. Louis, Missouri, United States; UCHealth University of Colorado Hospital (UCH), Aurora, Colorado, United States; Rutgers School of Public Health, Piscataway, New Jersey, United States; University of Colorado Anschutz Medical Campus, Aurora, Colorado, United States; University of Colorado Anschutz, Aurora, Colorado, United States; University of Colorado School of Medicine, Aurora, Colorado, United States

## Abstract

Many older patients have requested that a clinician be present during their medical aid in dying (MAID) procedure. Perceptions of organizational liability have led participating hospices to define the procedure as a sequence of key stages. Concerns that liability varies across stages has informed organizational restrictions regarding where in patient residences clinicians can be present. These “leave-the-room” policies have prompted dynamic—yet underexplored—patterns of clinician presence. This study aimed to map these trajectories. We used cross-sectional survey data from a convenience sample of hospice physicians, nurses, social workers, and chaplains. Eligible participants self-reported employment by a hospice that services states where MAID is legal and permits at least partial MAID participation (N = 100). Participants completed a matrix question inquiring about past presence. Rows indicated stages (during self-administration, after self-administration, after death). Columns indicated locations (in the same room, in the same residence but not the same room, never present). We consolidated responses to identify distinct trajectories, subsequently described via frequency distributions. Results revealed 13 distinct trajectories. The most common trajectories indicated never having been present (49%) and consistent presence in the room (24%). The 11 remaining trajectories reflected trends of increasing proximity to the patient (22%), increasing distance from the patient (2%), increasing proximity followed by increasing distance (2%), and consistent presence in the residence (1%). These 13 trajectories indicate substantial heterogeneity. Trends culminating in closer proximity suggest that perceived liability during self-administration declines across subsequent stages. Future studies should explore experiences of these trajectories alongside perceived quality of care.

